# The Structure Entropy-Based Node Importance Ranking Method for Graph Data

**DOI:** 10.3390/e25060941

**Published:** 2023-06-15

**Authors:** Shihu Liu, Haiyan Gao

**Affiliations:** School of Mathematics and Computer Science, Yunnan Minzu University, Kunming 650504, China; liush02@126.com

**Keywords:** graph data, node importance ranking, structure entropy

## Abstract

Due to its wide application across many disciplines, how to make an efficient ranking for nodes in graph data has become an urgent topic. It is well-known that most classical methods only consider the local structure information of nodes, but ignore the global structure information of graph data. In order to further explore the influence of structure information on node importance, this paper designs a structure entropy-based node importance ranking method. Firstly, the target node and its associated edges are removed from the initial graph data. Next, the structure entropy of graph data can be constructed by considering the local and global structure information at the same time, in which case all nodes can be ranked. The effectiveness of the proposed method was tested by comparing it with five benchmark methods. The experimental results show that the structure entropy-based node importance ranking method performs well on eight real-world datasets.

## 1. Introduction

As everyone knows, the key nodes usually play a decisive role during the process of graph data mining. In order to accurately identify the so-named key nodes in graph data, a priority problem is to construct an appropriate score function for ranking nodes [1,2,3,4]. Due to its prevalence in the field of disease detection [5,6], information transmission [7,8] and rumor blocking [9,10], how to rank nodes in graph data has been widely studied by researchers of various vocations.

In general, there are many traditional node importance ranking methods that only considered the local structure information of nodes to construct the score function [11,12,13]. For example, Lu et al. [14] calculated the importance of nodes by means of the degree centrality method. Chen et al. [15] constructed a multi-level neighbor information index to measure the importance of nodes, in which case only the degree information of first-order and second-order neighbors are considered. In order to distinguish the contribution of different neighbors, Katz [16] assigned different weights to them. The neighbors that can be reached by the short route are assigned the larger weight. At the same time, the neighbors that can be reached by the long route are assigned the small weight [17].

Up to now, many improved methods are proposed to deal with the problem of node importance ranking for graph data [18,19,20]. For instance, Freeman [21] constructed the betweenness centrality method, which described the importance of a node as the number of the shortest paths through it. In the closeness centrality method [22], the importance score of each node can be determined through the impact ability of the target node on other nodes. Based on the hypothesis that the node located at the core position has a strong influence, the K-shell decomposition method [23] is proposed. Yang et al. [24] proposed a comprehensive evaluation method based on multi-attribute decision making, which took many factors that affect the importance of nodes into account. What is more, the graph learning framework is also applied to evaluate the importance of nodes, such as in reference [25], the first graph neural network-based model is proposed to approximate betweenness and closeness centrality. Furthermore, Liu et al. [26] proposed a novel model based on self-supervised learning and graph convolution model to rank nodes, which formulated node importance ranking problem as a learning ranking problem.

Besides what has been discussed above, the well-known information entropy that has been proposed by Shannon [27] is also regarded as a powerful tool to measure the importance of nodes in a whole new perspective [28,29,30]. For instance, Zareie et al. [31] constructed the score function for each node, which considered the influence of neighbors on the target node with the help of information entropy. Guo et al. [32] proposed the EnRenew method by using the voting mechanism. In this method, information entropy is regarded as the voting ability of neighbors. By taking the effect of the spreading rate on information entropy into account, a propagation feature of the node-based ranking approach is introduced in reference [33]. Yu et al. [34] characterized the node importance as the node propagation entropy, which was the combination of degree and clustering coefficients.

Based on the above analysis, it can be found easily that in both the information entropy-based ranking methods and traditional ranking methods, only the local structure information is used to construct score functions. However, in fact, the global structure information, i.e., the connectivity of whole graph data, usually has a huge influence on the final ranking sequence [35,36,37]. In order to overcome the limitation or make full use of information from graph data, we propose a structural entropy-based node importance ranking method by considering the global structure information of graph data. We first calculate the amount of information contained in each connected component, which is denoted as the local structural entropy. Furthermore, the global structure entropy is constructed by distinguishing the different contributions of each connected component. Moreover, the effectiveness of the proposed method was tested on eight real-world datasets. The contribution of this paper can be listed as follows.

- The structure entropy-based node importance ranking method for graph data is proposed in terms of node removal.
- The local structural entropy is calculated by considering the degree of information of nodes and information entropy.
- The global structure entropy is constructed in terms of the connectivity of graph data.

The remainder of this paper is organized as follows. Section 2 reviews some basic concepts, which are graph data and benchmark methods for node importance ranking. Section 3 introduces the proposed method, i.e., the structural entropy-based node importance ranking method. Section 4 is composed of three parts, which are the experimental platform, datasets description and evaluation criteria. Section 5 shows the experimental results and contrastive analysis between the proposed method and five benchmark methods on eight real-world datasets. Section 6 is the summary of this paper and gives future research directions.

## 2. Preliminaries

In this section, some basic concepts are introduced, including the graph data and some benchmark methods for node importance ranking [38,39,40,41].

### 2.1. Graph Data

Generally speaking, the so-called graph data can be expressed as a tuple G=(V,E), where

- $V=\left\{v_{i}|i=1,2,\cdots ,n\right\}$ is the set of nodes and *n* represents the number of nodes.
- $E=\left\{\left(v_{i},v_{j}\right)|v_{i},v_{j}\in V\right\}$ is the set of edges and m=|E| represents the number of edges.

In this paper, we mainly discuss the undirected and unweighted graph data *G*. That is to say, $\left(v_{i},v_{j}\right)=\left(v_{j},v_{i}\right)$ for any $v_{i},v_{j}\in V$. Given that $v_{i},v_{j}\in V$, $(v_{i},v_{j})\in E$ if and only if there exists one edge that takes nodes $v_{i}$ and $v_{j}$ as its endpoint. For this situation, we use $a_{ij}=1$ to describe the fact that $v_{i}$ and $v_{j}$ are adjacent. Similarly, $a_{ij}=0$ denotes that $v_{i}$ and $v_{j}$ are not adjacent. With this representation, the adjacency of a given graph data *G* with *n* nodes is the following matrix

(1) $$A=\left(\begin{array}{cccc} a_{11} & a_{12} & \cdots & a_{1n}\\ a_{21} & a_{22} & \cdots & a_{2n}\\ \vdots & \vdots & \ddots & \vdots \\ a_{n1} & a_{n2} & \cdots & a_{nn} \end{array}\right).$$

### 2.2. Benchmark Methods

The key problem of node importance ranking is how to construct the score function. It is well-known that most classical methods apply local structure information of nodes to construct score functions. Some benchmark methods that can be used to rank the nodes are introduced in what follows.

#### 2.2.1. Degree Centrality Method

The degree centrality method (***DC***) determines the importance of node $v_{i}$ by the following equation

(2) $$DC\left(v_{i}\right)=d_{i},$$

where $d_{i}=\sum_{j=1}^{n}a_{ij}$ is the degree of node $v_{i}$.

#### 2.2.2. Closeness Centrality Method

The closeness centrality method (***CC***) defines the importance of node $v_{i}$ is

(3) $$CC\left(v_{i}\right)=\frac{1}{\sum_{i\neq j}d\left(i,j\right)},$$

where d(i,j) is the length of the shortest path from node $v_{i}$ to $v_{j}$, or $v_{j}$ to $v_{i}$.

#### 2.2.3. Improved K-Shell Decomposition Method

The classical K-shell decomposition method (***KS***) is a node removal-based method. A different Ks value that is regarded as the corresponding importance score is assigned to different nodes. In the first place, nodes with $d_{i}\leq 1$ are removed from the initial graph data *G*, and the same time value of Ks=1 is assigned to such nodes. After that, for the newly generated graph data, nodes with $d_{i}\leq 2,3,\cdots$, will be removed successively, in which case one will obtain the sequence Ks=2,3,⋯. For the improved K-shell decomposition method (***IKS***), it only removes nodes with the lowest degree each iteration. That is to say, the sequence of removed nodes is not based on the increasing sequence of degrees. For example, when all nodes with $d_{i}=2$ are removed, the node with $d_{i}=1$ may appear in the newly generated graph data. These nodes with $d_{i}=1$ will be removed next and obtain a higher IKs value.

#### 2.2.4. The Weight of Edges-Based Method

The weight of edges-based method (***WR***) determines the importance of node $v_{i}$ by the following equation

(4) $$WR\left(v_{i}\right)=\sum_{v_{j}\in N\left(v_{i}\right)}d_{i}d_{j},$$

where $N\left(v_{i}\right)=\left\{v_{j}|\left(v_{i},v_{j}\right)\in E\right\}$ is the set of neighbors of node $v_{i}$.

#### 2.2.5. The Gravity Model Based Method

Inspired by the thought of the classical gravity model, the gravity model-based method (***GM***) quantifies the importance of nodes by combining Ks value and shortest path information of nodes. The concrete calculation formula is

(5) $$GM\left(v_{i}\right)=\sum_{v_{j}\in \Psi \left(v_{i}\right)}\frac{Ks\left(v_{i}\right)Ks\left(v_{j}\right)}{d{\left(i,j\right)}^{2}},$$

where $\Psi (v_{i})$ is the set of nodes that defined by equation $\Psi \left(v_{i}\right)=\left\{v_{j}|d\left(i,j\right)\leq 3\right\}$.

## 3. The Proposed Method

It is well-known that most of the classical node importance ranking methods only consider the local structure information of nodes, but ignore the global structure information of graph data. For this, we combine the local and global structure information to construct the score function for all nodes. Based on the assumption that removing a more important node is likely to cause more structural variation of graph data, the score function is constructed from the perspective of node removal. Furthermore, the local and global structure information are considered comprehensively to construct the structure entropy of graph data and in which case all nodes can be ranked.

### 3.1. Node Removal

The graph data G=(V,E) are defined as a connected graph if there is a route from $v_{i}$ to $v_{j}$, or $v_{j}$ to $v_{i}$ for any nodes $v_{i},v_{j}\in G$. Otherwise, it is a disconnected graph. For a disconnected graph, each connected part is called a connected component.

**Figure 1:**
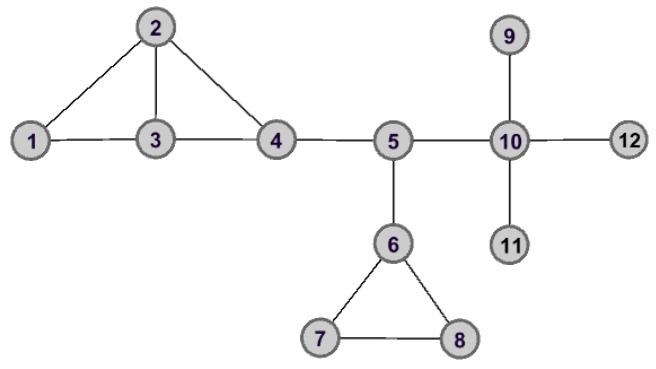
The connected graph with 12 nodes and 14 edges.

Taking Figure 1, for example, there are 12 nodes and 14 edges. One can find that the nodes $v_{3}$ and $v_{5}$ have the same degree. They will be assigned the same importance score according to the *DC* method. However, in fact, the importance of these two nodes is completely different.

**Figure 2:**
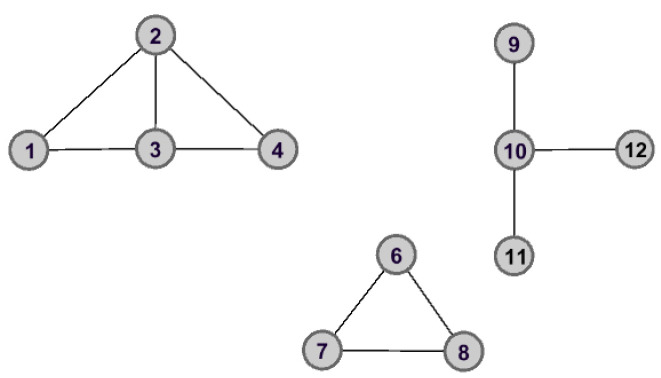
The graph data after removing node $v_{5}$.

**Figure 3:**
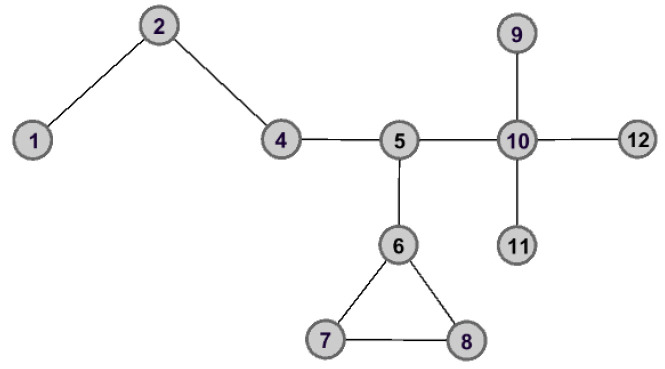
The graph data after removing node $v_{3}$.

As shown in Figure 2 and Figure 3, the graph data are divided into three connected components when node $v_{5}$ is removed. However, the removal of node $v_{3}$ does not lead to great changes for the structure of graph data, and the remaining graph data is still connected. Therefore, we can make the assertation that node $v_{5}$ would play a more important role than that of node $v_{3}$ in the aspect of structure information.

### 3.2. Local Structure Entropy

Removing the important node may lead to the fact that the graph data will be divided into more than one connected component. In order to quantify the global structure information of graph data reasonably, calculating the amount of information about each connected component is a priority problem. Hereinto, we first construct the local structure entropy for each connected component with the help of information entropy.

The information entropy is usually used to measure the amount of information about an event. For the random variable $X=(x_{1},x_{2},\cdots ,x_{n})$, given that its probability distribution is $P=(p_{1},p_{2},\cdots ,p_{n})$, then the information entropy of *X* is

(6) $$E\left(X\right)=-\sum_{i=1}^{n}p_{i}log_{2}p_{i}.$$

Following Equation (6), one can find that the more likely an event is to happen, the less information it contains, and vice versa. Once some nodes are removed, the graph data will be changed into more than one connected component with a high probability. This will lead to information decreasing of the corresponding connected component. That is to say, the appearance of connected components is a frequent event and it contains less structure information. This is consistent with the property of information entropy. Therefore, the amount of structure information contained in each connected component can be quantified by information entropy, and it can be defined as local structural entropy. In what follows, we give a detailed description of local structural entropy.

Given that G=(V,E) is graph data with *n* nodes and *m* edges. The initial graph data are divided into *s* connected components after removing the target node from *G*, denoted as $C_{1},C_{2},\cdots ,C_{s}$. Each connected component contains $|C_{i}|$ nodes, for i=1,2,⋯,s. Then, the probability distribution $P(C_{i})$, for i=1,2,⋯,s, can be expressed as

(7) $$P\left(C_{i}\right)=\left(p\left(v_{1}\right),p\left(v_{2}\right),\cdots ,p\left(v_{|C_{i}|}\right)\right),$$

where

(8) $$p\left(v_{t}\right)=\left\{\begin{array}{cc} \frac{\sum_{v_{j}\in N\left(v_{t}\right)}d_{j}}{\sum_{v_{x}\in C_{i}}d_{x}^{2}}, & \;\;|C_{i}|>1\\ \; & \;\\ 1, & \;\;|C_{i}|=1 \end{array}\right.$$

for $t=1,2,\cdots ,|C_{i}|$. Obviously, this probability distribution satisfies the constraint that the sum of probability is equal to 1 for each connected component, i.e., $\sum_{v_{t}\in C_{i}}p\left(v_{t}\right)=1$.

According to Equation (6), the local structure entropy with respect to the connected component $C_{i}$, for i=1,2,⋯,s, can be defined as

(9) $$LE\left(C_{i}\right)=-\sum_{v_{j}\in C_{i}}p\left(v_{j}\right)log_{2}p\left(v_{j}\right).$$

It can be easily found that Equation (9) has the following properties.

**Property** **1.** *Given that G is graph data and $C_{i}$ is the ith connected component of G by removing $v_{i}$ from G. Then, one has that $LE(C_{i})\geq 0$.*

**Proof.**  If $|C_{i}|=1$, taking $v_{t}\in C_{i}$ for example, then $p(v_{t})=1$. To this

(10) $$\begin{array}{cc} LE(C_{i}) & =-\sum_{v_{j}\in C_{i}}p\left(v_{j}\right)log_{2}p\left(v_{j}\right)\\ \; & =p\left(v_{t}\right)log_{2}p\left(v_{t}\right)\\ \; & =0. \end{array}$$

If $|C_{i}|>1$, one has that $p\left(v_{j}\right)log_{2}p\left(v_{j}\right)<0$ for any $v_{j}\in C_{i}$, then

(11) $$\begin{array}{cc} LE(C_{i}) & =-\sum_{v_{j}\in C_{i}}p\left(v_{j}\right)log_{2}p\left(v_{j}\right)\\ \; & >0. \end{array}$$

This completes the proof.    □

**Property** **2.** *Given that G is a graph data and $C_{i}$ is the ith connected component of G by removing $v_{i}$ from G. Then, the value of $LE(C_{i})$ is not relevant to the position of $p(v_{t})$ in $P(C_{i})$, for $v_{t}\in C_{i}$.*

**Proof.**  For the connected component $C_{i}$, the initial probability distribution is $P\left(C_{i}\right)=\left(p\left(v_{1}\right),p\left(v_{2}\right),\cdots ,p\left(v_{|C_{i}|}\right)\right)$. If $p(v_{1})$ and $p(v_{2})$ change the position, the probability distribution changes into $P\left({\bar{C}}_{i}\right)=\left(p\left(v_{2}\right),p\left(v_{1}\right),\cdots ,p\left(v_{|C_{i}|}\right)\right)$.

With the help of Equation (9), the following result

(12) $$\begin{array}{cc} LE(C_{i}) & =-\sum_{v_{j}\in C_{i}}p\left(v_{j}\right)log_{2}p\left(v_{j}\right)\\ \; & =-\left(p\left(v_{1}\right)log_{2}p\left(v_{1}\right)+p\left(v_{2}\right)log_{2}p\left(v_{2}\right)\right)-\sum_{v_{j}\in C_{i},v_{j}\neq v_{1},v_{2}}p\left(v_{j}\right)log_{2}p\left(v_{j}\right)\\ \; & =-\left(p\left(v_{2}\right)log_{2}p\left(v_{2}\right)+p\left(v_{1}\right)log_{2}p\left(v_{1}\right)\right)-\sum_{v_{j}\in C_{i},v_{j}\neq v_{1},v_{2}}p\left(v_{j}\right)log_{2}p\left(v_{j}\right)\\ \; & =LE({\bar{C}}_{i}) \end{array}$$

comes naturally.

This completes the proof.    □

**Property** **3.** *Given that $C_{i}$ and $C_{j}$, respectively, are the ith and jth connected components of G by removing node $v_{i}$ from G. Then, their overall structure entropy can be expressed as the sum of local structure entropy, i.e., $LE\left(C_{i}C_{j}\right)=LE\left(C_{i}\right)+LE\left(C_{j}\right)$.*

**Proof.**  According to the Equations (7) and (8), the probability distributions of $C_{i}$ and $C_{j}$ is

(13) $$P\left(C_{i}\right)=\left(p\left(v_{1}\right),p\left(v_{2}\right),\cdots ,p\left(v_{|C_{i}|}\right)\right)$$

and

(14) $$P\left(C_{j}\right)=\left(p\left(v_{1}^{\prime }\right),p\left(v_{2}^{\prime }\right),\cdots ,p\left(v_{|C_{j}|}^{\prime }\right)\right),$$

where $\sum_{v_{t}\in C_{i}}p\left(v_{t}\right)=1$, for $t=1,2,\cdots ,|C_{i}|$ and $\sum_{v_{x}^{\prime }\in C_{j}}p\left(v_{x}^{\prime }\right)=1$, for $x=1,2,\cdots ,|C_{j}|$.

For independent connected components $C_{i}$ and $C_{j}$, their joint probability distribution can be expressed as

$$P\left(C_{i}C_{j}\right)=\left(p\left(v_{1}\right)p\left(v_{1}^{\prime }\right),p\left(v_{1}\right)p\left(v_{2}^{\prime }\right),\cdots ,p\left(v_{1}\right)p\left(v_{|C_{j}|}^{\prime }\right),p\left(v_{2}\right)p\left(v_{1}^{\prime }\right),\cdots ,p\left(v_{|C_{i}|}\right)p\left(v_{|C_{j}|}^{\prime }\right)\right),$$

where $\sum_{v_{t}\in C_{i}}\sum_{v_{x}^{\prime }\in C_{j}}p\left(v_{t}\right)p\left(v_{x}^{\prime }\right)=1$, for $t=1,2,\cdots ,|C_{i}|$ and $x=1,2,\cdots ,|C_{j}|$.

With the help of Equation (9), one can have that

(15) $$\begin{array}{cc} LE(C_{i}C_{j}) & =-\sum_{v_{t}\in C_{i}}\sum_{v_{x}^{\prime }\in C_{j}}p\left(v_{t}\right)p\left(v_{x}^{\prime }\right)log_{2}\left(p\left(v_{t}\right)p\left(v_{x}^{\prime }\right)\right)\\ \; & =-\sum_{v_{t}\in C_{i}}\sum_{v_{x}^{\prime }\in C_{j}}p\left(v_{t}\right)p\left(v_{x}^{\prime }\right)log_{2}p\left(v_{t}\right)-\sum_{v_{t}\in C_{i}}\sum_{v_{x}^{\prime }\in C_{j}}p\left(v_{t}\right)p\left(v_{x}^{\prime }\right)log_{2}p\left(v_{x}^{\prime }\right)\\ \; & =-\sum_{v_{x}^{\prime }\in C_{j}}p\left(v_{x}^{\prime }\right)\sum_{v_{t}\in C_{i}}p\left(v_{t}\right)log_{2}p\left(v_{t}\right)-\sum_{v_{t}\in C_{i}}p\left(v_{t}\right)\sum_{v_{x}^{\prime }\in C_{j}}p\left(v_{x}^{\prime }\right)log_{2}p\left(v_{x}^{\prime }\right)\\ \; & =LE\left(C_{i}\right)+LE\left(C_{j}\right). \end{array}$$

This completes the proof.    □

### 3.3. Global Structure Entropy

The key problem in this section is to quantify the information contained in the whole graph data. According to Property 3, one can find that the overall structure entropy of *G* can be expressed as the sum of local structure entropy about each connected component. In order to distinguish the contribution of different connected components, in what follows, we take the number of edges as the weight value of each local structure entropy.

Given that *G* is graph data and taking node $v_{i}\in V$ as an example, the global structure entropy of *G* is quantified by combining the number of edges and local structure entropy, which can be defined as

(16) $$SE\left(v_{i}\right)=\sum_{j=1}^{s}\left|E_{j}\right|LE\left(C_{j}\right),$$

where $|E_{j}|$ is the number of edges in each connected component, for j=1,2,⋯,s.

The information contained in graph data *G* will decrease if the more important node $v_{i}$ is removed. That is to say, the global structure entropy will get the smaller value of $SE(v_{i})$. Therefore, the global structure entropy can be regarded as a cost function. In other words, the smaller the value of $SE(v_{i})$, the more important the node $v_{i}$. For this, one can obtain a possible sequence, such as $v_{i1}≽v_{i2}≽\cdots ≽v_{in}$, where (i1,i2,⋯,in) is a certain permutation of (1,2,⋯,n). For example, $v_{i1}≽v_{i2}$ if and only if $SE\left(v_{i1}\right)\leq SE\left(v_{i2}\right)$, and $v_{i1}≺v_{i2}$ if and only if $SE\left(v_{i1}\right)>SE\left(v_{i2}\right)$.

**Example** **1.** *To make it easy to understand how to calculate the global structure entropy of each node, in what follows, we apply a simple graph data G shown in Figure 1 to describe the whole process in detail.*

Taking node $v_{5}$ for example, the initial graph data is divided into three connected components after removing node $v_{5}$ from *G*, which are $C_{1},C_{2}$ and $C_{3}$. With the help of Equations (7) and (8), the probability distribution of connected components can be determined, which are

$$P\left(C_{1}\right)=\left(\frac{6}{26},\frac{7}{26},\frac{7}{26},\frac{6}{26}\right)$$

$$P\left(C_{2}\right)=\left(\frac{4}{12},\frac{4}{12},\frac{4}{12}\right)$$

and

$$P\left(C_{3}\right)=\left(\frac{3}{12},\frac{3}{12},\frac{3}{12}\right).$$

Then, the local structure entropy of each connected component could be obtained by Equation (9), which are

$$LE(C_{1})=1.9958$$

$$LE(C_{2})=1.5849$$

and

$$LE(C_{3})=2.0000.$$

With Equation (16), the global structure entropy is

(17) $$\begin{array}{cc} SE(v_{5}) & =\sum_{j=1}^{3}\left|E_{j}\right|LE\left(C_{j}\right)\\ \; & =20.7337. \end{array}$$

The calculation of other nodes is the same as that of $v_{5}$. Here, we list the top six nodes in Table 1.

As can be seen from Table 1, one has that $SE\left(v_{5}\right)<SE\left(v_{3}\right)$, then their importance can be ranked as $v_{5}≻v_{3}$. It is worth mentioning that this is consistent with the analysis results in Section 3.1.

### 3.4. Algorithm Description

Bearing what was discussed in mind, we give the detailed process of the structure entropy-based node importance ranking method for graph data *G* in Algorithm 1. For convenience, here we apply the abbreviation ***SE*** to represent the proposed method.    
**Algorithm 1:** The *SE* method.
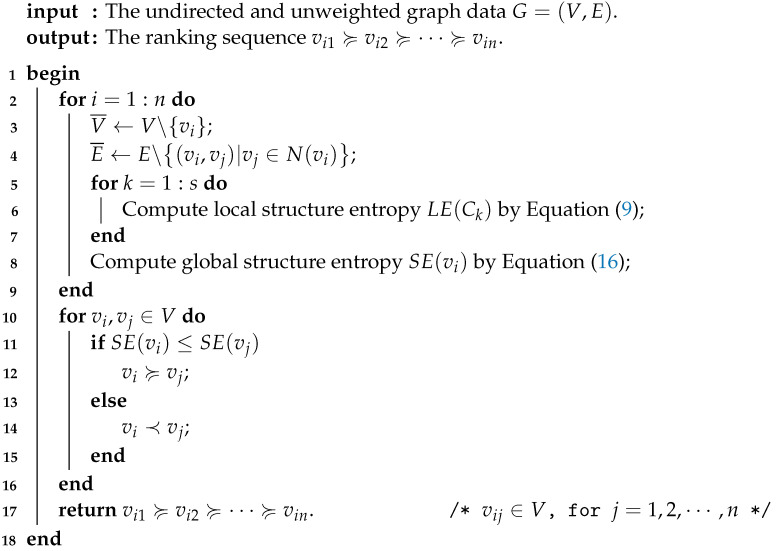


## 4. Experimental Construction

In this section, we introduce the experimental platform, experimental datasets and evaluation criteria.

### 4.1. Experimental Platform

The algorithm development platform is MATLAB R2018a. The computer configuration used for the experiment is the following: Intel(R)Core(TM)i5-8250U CPU, 8 GB installed memory and 64-bit Windows 10 operating system.

### 4.2. Datasets Description

From the website http://konect.cc/networks/ (accessed on 10 April 2023), we downloaded the eight real-world datasets for experimental analysis. The detailed information on these datasets is given below.

- **Contiguous USA (*CONT*)**: The network of shared border between 48 contiguous states.
- **Les Miserables (*LESM*)**: The network of co-appearances of characters in the novel “Les Miserables”.
- **Polbooks (*POLB*):** The network of books about US politics published in 2004.
- **Adjnoun (*ADJN*)**: The network of co-words between adjectives and nouns commonly used in the novel “David Copperfield”.
- **Football (*FOOT*)**: The network of US football games between division IA colleges.
- **Netscience (*NETS*)**: The collaborative network of scientists who have published papers in the field of network science.
- **Email (*EMAI*)**: The interactive network of emails between members in University of Rovira.
- **Hamsterster households (*HAMS*)**: The network of family relationships between members using the same website.

Table 2 shows the topological statistical information of the above eight real-world datasets, where <d> is the average degree, $d_{max}$ is the maximum degree and cc is the average clustering coefficient of datasets ($cc=\frac{1}{n}\sum_{i=1}^{n}c_{i}$, where $c_{i}$ is the local clustering coefficient of node $v_{i}$ and $c_{i}=\frac{\sum_{v_{j}\in N\left(v_{i}\right)}d_{j}}{d_{i}\left(d_{i}-1\right)}$).

As shown in Table 2, the eight real-world datasets used for the experimental analysis have the following different properties. The number of nodes in *CONT* and *LESM* datasets are both less than 100, which is mainly used to verify the effectiveness of the proposed method on small-scale datasets. Although *POLB*, *ADJN* and *FOOT* datasets have similar scale, <d> and $d_{max}$ of the *FOOT* dataset are very close. This indicates the fact that there are a large number of nodes with the same degree in the *FOOT* dataset. Since the *NETS* dataset has the largest cc in all datasets, the distribution of nodes is dense. The *EMAI* and *HAMS* belong to the larger-scale datasets. Hereinto, the *EMAI* is the dataset with the highest number of edges. The *HAMS* dataset has the highest number of nodes and the smallest average clustering coefficient in all datasets. In fact, the biggest difference between the *HAMS* and other datasets is that it contains 655 isolated nodes. The extensibility of ranking methods can be reflected in this kind of special dataset.

### 4.3. Evaluation Criteria

Here, we introduce four evaluation criteria to verify the validity of the proposed method. The more detailed information can be found in the literature [42,43,44,45].

#### 4.3.1. Monotonicity-Based Evaluation Criterion

It is well-known that the fewer nodes that obtain the same importance score, the better the corresponding ranking method. Here, the discriminability of the proposed method can be evaluated by using the monotonicity relation function. Its mathematical formula is

(18) $$M\left(R\right)={\left(1-\frac{\sum_{r\in \Gamma }n_{r}{\left(n_{r}-1\right)}^{2}}{n(n-1)}\right)}^{2},$$

where *R* is the final ranking sequence, Γ is the index set that represents different orders in the ranking sequence *R*, r∈Γ and $n_{r}$ represents the number of nodes that have been listed in the same order. For example, if the ranking sequence *R* is $v_{1}≻v_{2}\approx v_{3}≻v_{4}$, then $\Gamma =\left\{1,2,3\right\}$ and $n_{1}=n_{3}=1$ and $n_{2}=2$. Obviously, if all nodes have the same order in the ranking sequence *R*, then the value of M(R) is 0. If each node can obtain a unique order, the value of M(R) is 1 and the ranking sequence *R* is completely monotonic.

#### 4.3.2. Complementary Cumulative Distribution Function Based Evaluation Criterion

In addition to monotonicity, the complementary cumulative distribution function (***CCDF***) is utilized to further evaluate the discriminability of the proposed method. Its mathematical expression is

(19) $$CCDF\left(r\right)=\frac{n-\sum_{i=1}^{r}n_{i}}{n}.$$

Obviously, with the increasing of *r*, if more nodes are assigned to the same order, then the value of the function will decrease rapidly, until to 0.

#### 4.3.3. Connected Component Based Evaluation Criterion

Generally, the robustness of the ranking method can be quantified by the deliberate attack strategy. Firstly, some nodes are removed from graph data *G* according to the ranking sequence *R*, which can decrease the connectivity of *G*. After that, the robustness of the ranking method is evaluated from two perspectives, i.e., the number of connected components and the proportion of the maximum connected component. The former can be expressed as ξ, and the definition of latter is

(20) $$\tau =\frac{M_{s}}{n},$$

where

(21) $$M_{s}=max\left\{|C_{1}\left|,\right|C_{2}\left|,\cdots ,\right|C_{s}|\right\}$$

represents the number of nodes that are contained in the maximum connected component. Obviously, one can find that the larger value of ξ and the smaller value of τ, the stronger the robustness of the corresponding ranking method.

#### 4.3.4. Susceptible-Infected-Recovered Epidemic Model-Based Evaluation Criterion

The accuracy of different ranking methods can be verified by using the Susceptible-Infected-Recovered epidemic model (***SIR***). Nodes in *SIR* are classified into infected state, susceptible state, and recovered state. In the whole process of infection, the initial infected node can affect its neighbors with the infected probability β, and enter into a recovered state with the recovery probability γ. Nodes that are already in the recovery state will not participate in the subsequent infection process. To increase accuracy, the experiment will repeat hundreds of times and the average number of infected nodes is taken as the propagation ability of the seed node, denoted as F(R). Its calculation formula is defined as

(22) $$F\left(R\right)=\frac{n_{I}}{N_{ite}},$$

where $n_{I}$ is the number of nodes infected by seeds and $N_{ite}$ is the number of repeated experiments.

## 5. Results and Analysis

In this section, the performance of the proposed method *SE* is demonstrated on eight real-world datasets. In order to show the results more clearly, all datasets are classified into three classes in the aspect of the number of nodes, i.e., the datasets *CONT* and *LESM* with n≤100, the datasets *POLB*, *ADJN*, *FOOT* and *NETS* with 100<n≤1000, the datasets *EMAI* and *HAMS* with n>1000.

### 5.1. Monotonicity Analysis

In this part, we analyze the effectiveness of *SE* by comparing the monotonicity of ranking sequence *R* obtained by *SE* with other benchmark methods. Table 3 shows the value of monotonicity under *DC*, *CC*, *IKS*, *WR*, *GM* and *SE* methods. One can find that the *SE* method can obtain the maximum monotonicity value on all datasets. Obviously, this advantage is independent of the number of nodes.

#### 5.1.1. On *CONT* and *LESM* Datasets

From Table 3, one can find that for the *CONT* dataset, all methods except *DC* and *IKS*, the monotonicity is greater than 0.9000. The main reason is that the two methods, i.e., *DC* and *IKS* methods, can be influenced easily by the degree of nodes. It is worth mentioning that the *SE* method is less affected by the degree of information about nodes. Therefore, it is superior to *DC* and *IKS* methods in monotonicity.

On the *LESM* dataset, it should be pointed out that both *SE* and *GM* methods can achieve the maximum value of monotonicity at the same time. From Table 2, one can find that the *LESM* dataset has a higher cc value in datasets with a similar number of nodes. For datasets with dense distribution of nodes, the method that the structure information of nodes is considered during the ranking procedure can identify the importance of nodes more efficiently, such as *SE* and *GM* methods. This also confirms that the *SE* method has great merit on small-scale datasets.

#### 5.1.2. On *POLB*, *ADJN*, *FOOT* and *NETS* Datasets

Since the *POLB*, *ADJN* and *FOOT* datasets have similar scales, most methods achieve similar monotonicity. In this case, the *SE* method still shows obvious advantages. One can observe that the *SE* method not only obtains the highest monotonicity value on all datasets but also assigns the unique order to each node on *POLB* and *FOOT* datasets. Table 2 shows that <d> and $d_{max}$ of the *FOOT* dataset are very close. This indicates the fact that there are a large number of nodes with the same degree in the *FOOT* dataset. Since the *DC* method have no ability to identify the importance of these nodes, it achieves the worst monotonicity. On the contrary, the *SE* method can obtain a completely monotonous ranking sequence. What is more, the difference in monotonicity value between *SE* and *DC* methods is as high as 0.6464. For this, we can guess that the *SE* method would show better performance on large-scale graph data.

On the *NETS* dataset, the *CC* and *GM* methods obtain similar monotonicity values, but *IKS* is still the worst-performing method. Since the *NETS* dataset has the largest cc in all datasets, the distribution of nodes is dense. Obviously, the *IKS* method has the worst performance on this dataset. The main reason is that the *IKS* method mainly considers the location information of nodes largely, and usually treats nodes with adjacent locations as equally important. On the contrary, the *SE* method is not affected by the location of nodes and can still obtain the maximum value of monotonicity.

#### 5.1.3. On *EMAI* and *HAMS* Datasets

For datasets with a large number of nodes, such as the *EMAI* and *HAMS* datasets, one can find that the *GM* method shows the same advantage as the *SE* method and the performance of *CC* and *WR* methods also increases. As shown in Table 2, the *HAMS* dataset has the highest number of nodes and the smallest clustering coefficient in all datasets, which indicates that the nodes in the *HAMS* dataset are more dispersed. In fact, the biggest difference between the *HAMS* and other datasets is that it contains 655 isolated nodes. Due to this special structure, the importance of most nodes cannot be identified on the *HAMS* dataset. However, the *SE* method still obtains the maximum value of monotonicity. This further verifies the effectiveness of the *SE* method on datasets with special structure.

### 5.2. Node Distribution Analysis

**Figure 4:**
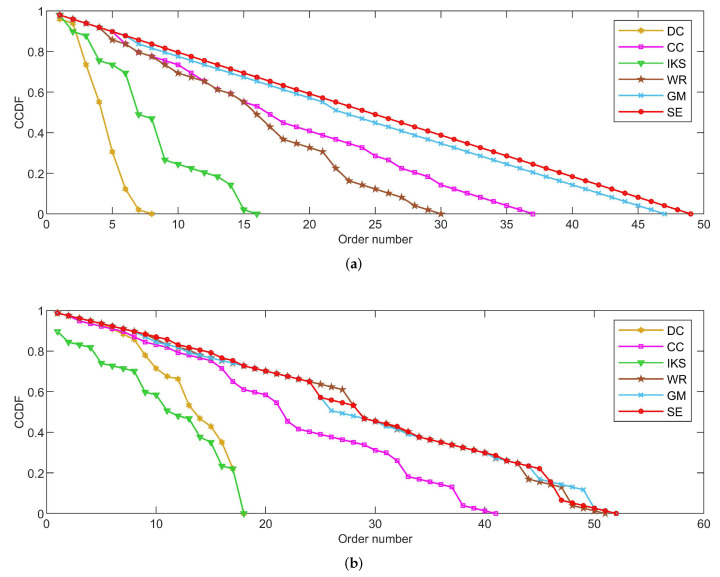
The curves of *CCDF* on (**a**) *CONT* and (**b**) *LESM* datasets.

**Figure 5:**
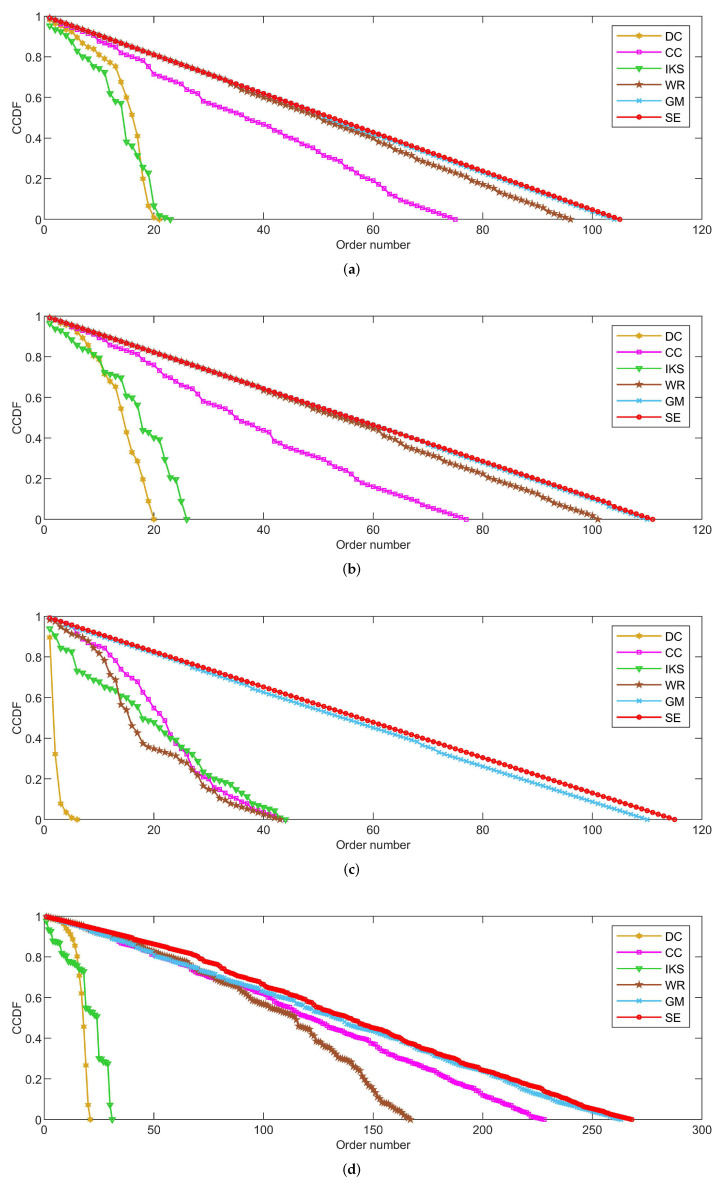
The curves of *CCDF* on (**a**) *POLB*; (**b**) *ADJN*; (**c**) *FOOT*; and (**d**) *NETS* datasets.

**Figure 6:**
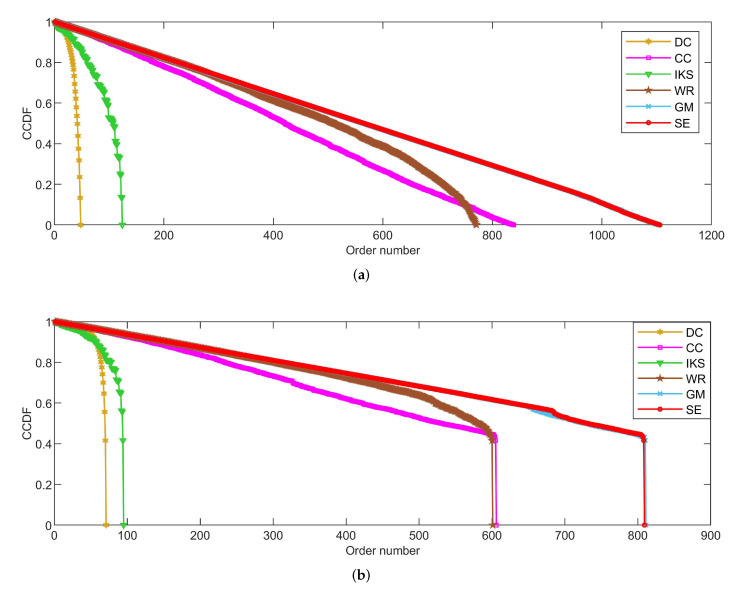
The curves of *CCDF* on (**a**) *EMAI* and (**b**) *HAMS* datasets.

As shown in Figure 4, Figure 5 and Figure 6, the *CCDF* curves express the node distribution of the ranking sequence obtained by different methods. Here, we mainly focus on two perspectives. On the one hand, the descending slope of curves can indicate the discriminability of the corresponding method. The method with the smoother descending slope can distribute the fewer nodes in the same order. On the other hand, we focus on the value of the horizontal axis when the value of the vertical axis is equal to 0, which can represent the total order number that can be generated by the corresponding method. The larger the order number, the better the discriminability of the corresponding method.

From the results, it can be found that the *SE* method can obtain the smoother descending slope and the maximum order number on most datasets. That is to say, the *SE* method should distribute the fewer nodes to the same order and more clearly identify the importance of different nodes compared with benchmark methods.

#### 5.2.1. On *CONT* and *LESM* Datasets

Figure 4 is the curves of *CCDF* on *CONT* and *LESM* datasets. Obviously, the *SE* method obtains the smoothest descending slope and descends keeping in a straight line as shown in Figure 4a. What is more, the total order number obtained by the *SE* method is 49, which is equivalent to the node number of *CONT* dataset. In other words, only one node is located at the corresponding location of the ranking sequence. Nicely, this is consistent with that of Table 3.

From Figure 4b, it can be easily found that both *GM* and *SE* methods obtain the maximum order number 52 at the same time. However, that of *DC* and *IKS* methods is 18, which means that there are 59 nodes whose importance cannot be identified. Such defects are more evident on larger-scale datasets and this can be confirmed by the following experiments. In addition, although there is no method that can completely identify the importance of all nodes, the *SE* method shows greater advantage when the order number is between 10 and 20.

#### 5.2.2. On *POLB*, *ADJN*, *FOOT* and *NETS* Datasets

Figure 5 is the curves of *CCDF* on *POLB*, *ADJN*, *FOOT* and *NETS* datasets. As can be seen, the advantage of the *SE* method is obvious. On the one hand, the *SE* method achieves the maximum order number on all datasets. This reflects the fact that the *SE* method can distribute fewer nodes to the same order compared with other benchmark methods. On the other hand, the *SE* method obtains the smoothest descending slope in all of the comparison methods. Especially on the *POLB* and *FOOT* datasets, *SE* is the only method that can descend keeping in a straight line. For this, we can guess that the *SE* method would show better performance on large-scale datasets.

On the whole, the performance of *DC* and *IKS* methods is relatively poor. The order number obtained by *DC* and *IKS* methods is only 10% to 20% of the total number of nodes. This means that nearly 80% to 90% of nodes’ importance cannot be identified. Frankly speaking, they cannot be regarded as good ranking methods.

#### 5.2.3. On *EMAI* and *HAMS* Datasets

Figure 6 is the curves of *CCDF* for two large-scale datasets, i.e., the *EMAI* and *HAMS* datasets. As can be seen, the performance of all methods can be divided into three categories roughly, i.e., well-performing *SE* and *GM* methods, moderately-performing *CC* and *WR* methods, and poorly-performing *DC* and *IKS* methods. Especially in Figure 6b, it should be pointed out that the curves of *CCDF* obtained by all methods have a process of vertical decline. The main reason for this phenomenon is that it is difficult to identify the importance of isolated nodes. Here, the importance score of an isolated node is set to the minimum in all methods. Although the importance of isolated nodes is not significant, this special structure can affect the importance scores of other nodes. From Figure 6b, it can be easily seen that the *SE* method still obtains the smoothest descending slope in all comparison methods. What is more, the biggest difference in total order numbers between *SE* and other methods can exceed 700. This further validates the effectiveness of *SE* method proposed in this paper.

### 5.3. Robustness Analysis

In this subsection, we evaluate the robustness of the SE method by comparing the curves of ξ and τ of *SE* method with that of other benchmark methods.

**Figure 7:**
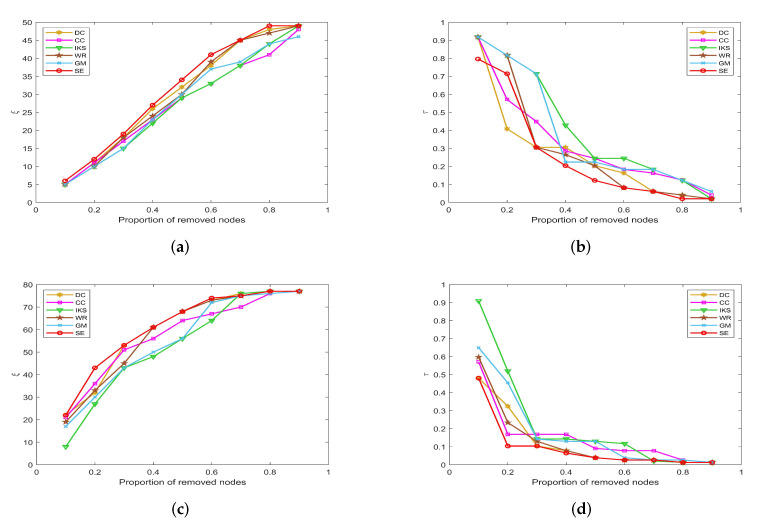
The curves of ξ and τ on (**a**,**b**) *CONT* and (**c**,**d**) *LESM* datasets.

**Figure 8:**
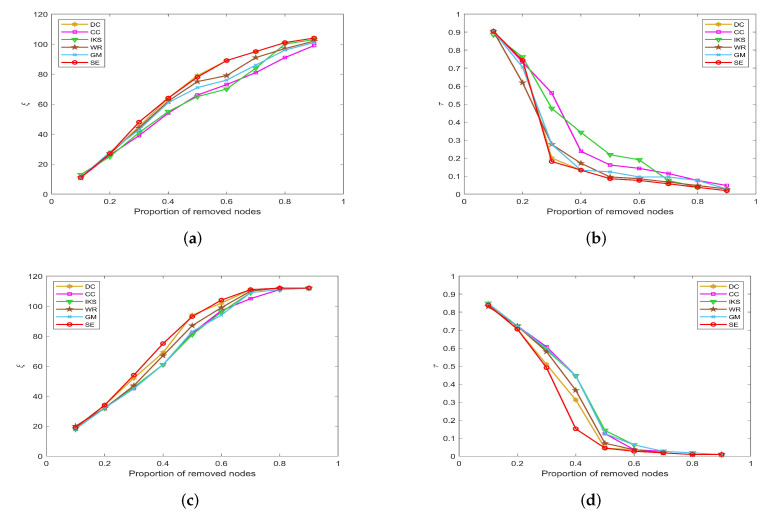
The curves of ξ and τ on (**a**,**b**) *POLB* and (**c**,**d**) *ADJN* datasets.

**Figure 9:**
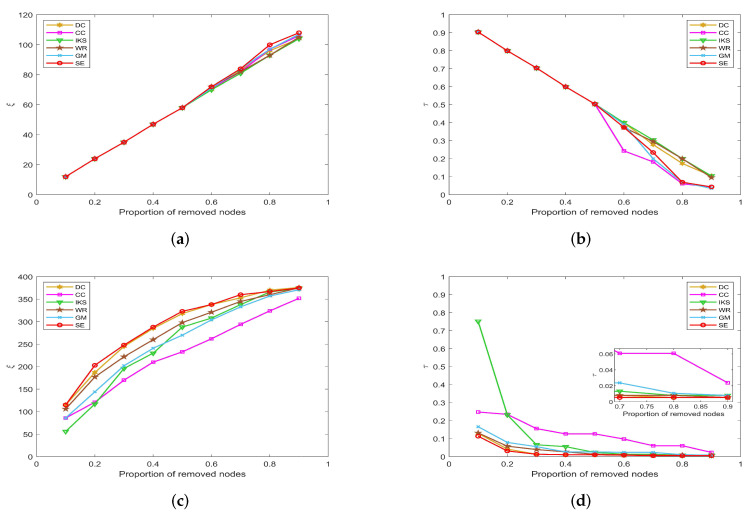
The curves of ξ and τ on (**a**,**b**) *FOOT* and (**c**,**d**) *NETS* datasets.

**Figure 10:**
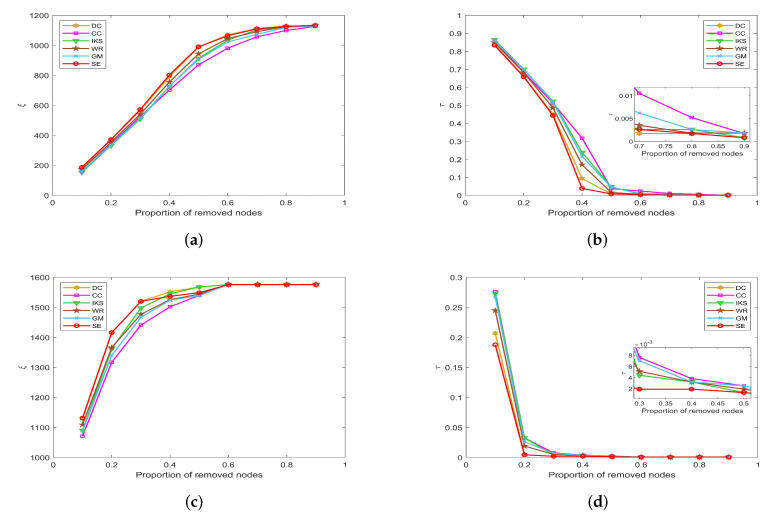
The curves of ξ and τ on (**a**,**b**) *EMAI* and (**c**,**d**) *HAMS* datasets.

- The left side of Figure 7, Figure 8, Figure 9 and Figure 10 is the curves of ξ, which shows the number changes of connected components. The horizontal axis of subfigures represents the proportion of removed nodes and the vertical axis represents the number of connected components after removing nodes from the dataset.
- The right side of Figure 7, Figure 8, Figure 9 and Figure 10 is the curves of τ, which shows the variation of the maximum connected component. The horizontal axis of subfigures represents the proportion of removed nodes and the vertical axis represents the value of τ calculated by Equation (20).

The larger value of ξ and the smaller value of τ, the stronger the robustness of the corresponding ranking method. From the result, it can be found that the ξ curves of *SE* method obtain the faster uptrend and the τ curves of *SE* method obtain the faster downtrend in most datasets as the proportion of removed nodes increases.

#### 5.3.1. On *CONT* and *LESM* Datasets

Figure 7 is the curves of ξ and τ on *CONT* and *LESM* datasets. As can be seen from Figure 7a, when the proportion of removed nodes changes from 10% to 90%, the *SE* method can always obtain the maximum value of ξ. Obviously, the *SE* method has the most obvious upward trend compared with other methods. In Figure 7b, when the proportion of removed nodes is only 10%, the τ value of all methods is equal to 0.9184 except *SE* method. In fact, the value of τ obtained by *SE* method only is 0.7959, which is 0.1225 lower than other methods. This advantage is more pronounced after the proportion of removed nodes reaches 30%.

On the *LESM* dataset, as shown in Figure 7c,d, the differences between the six methods are obvious. Especially when the proportion of removed nodes is 20%, the value of ξ corresponding to *DC*, *CC*, *IKS*, *WR*, *GM* and *SE* methods is 32, 36, 27, 33, 30 and 43, respectively. Obviously, the *SE* method is superior to other methods. What is more, the value of τ corresponding to the above methods is 0.3247, 0.1688, 0.5193, 0.2338, 0.4545 and 0.1039, respectively. One can find that the difference between the *SE* and *IKS* method is as high as 0.4154. That is to say, the maximum connected component of the *IKS* method contains 40 nodes, while that of the *SE* method contains only 8 nodes. This fully confirms that *SE* method has better robustness in small-scale datasets.

#### 5.3.2. On *POLB*, *ADJN*, *FOOT* and *NETS* Datasets

Figure 8 is the curves of ξ and τ on *POLB* and *ADJN* datasets. With the increase in dataset scale, the robustness of the *CC* method decreases significantly, but the advantage of the *DC* method becomes more obvious. As shown in Figure 8a,c, both *DC* and *SE* methods obtain the same value of ξ in most cases. The main reason is that the *DC* method regards the nodes with larger degrees as the more important nodes, and these nodes can affect the number of connected components to a great extent. On the whole, the robustness of the *IKS* method is relatively poor. From Figure 8b,d, it can be found that the curves of τ corresponding to the *SE* method can maintain the fastest downtrend when the proportion of removed nodes starts from 30%.

Figure 9 is the curves of ξ and τ on *FOOT* and *NETS* datasets. One can observe that all methods obtain similar ranking sequences on *FOOT* dataset. As shown in Figure 9a,b, six ranking methods show the same robustness until the proportion of removed nodes is as high as 50%. However, in fact, when the proportion of removed nodes is greater than 50%, the *SE* method obtains the largest value of ξ, and the *CC* method obtains the smallest value of τ.

It is a pity that the *CC* method does not show better robustness on datasets with more nodes, such as the *NETS* dataset. From Figure 9c, it can be found that the value of ξ obtained by the *CC* method is much lower than that of other methods, and the maximum difference between *CC* and *SE* methods reaches as high as 90. Similarly, as shown in Figure 9d, the value of τ corresponding to the *CC* method is much higher than other methods, and the maximum difference between *CC* and *SE* methods is as high as 0.2031. For this, we can guess that the *SE* method would show more excellent robustness on large-scale dataset.

#### 5.3.3. On *EMAI* and *HAMS* Datasets

Figure 10 is the curves of ξ and τ on *EMAI* and *HAMS* datasets. As shown in Figure 10a,b, the *SE* and *DC* methods can maintain absolute superiority compared with other benchmark methods. Table 2 shows that the *EMAI* dataset has the largest value of *m* and <d> is as high as 9.6230. For this kind of tightly connected large-scale dataset, the *CC* and *IKS* methods perform poorly, and WE and *GM* methods are always in the middle position. The advantages of *DC* and *SE* methods are not easy to distinguish. It should be pointed out that all methods are close to the minimum value of τ when the proportion of removed nodes is greater than 40%. However, in fact, when the proportion of removed nodes is equal to 40%, the *SE* method is significantly better than other methods. This fully confirms that the *SE* method has better robustness compared with other benchmark methods.

By observing Figure 10c,d, the value of ξ and τ will not change after the proportion of removed nodes is greater than 60%. The reason is that the *HAMS* dataset contains 655 isolated nodes, which is more than 40% of the total number of nodes. However, in fact, the *SE* method can obtain the minimum value of τ when the proportion of removed nodes is between 10% and 50%. This further verifies that the *SE* method is also more robust for datasets with special structures.

### 5.4. Accuracy Analysis

In this part, we mainly analyze the accuracy of the *SE* method to identify key nodes in terms of the *SIR* model. Herein, we select the top 2, 4, 6, 8 and 10 nodes listed in the front of the ranking sequence as seeds for datasets with n≤1000. For datasets with more than 1000 nodes, we select top 20, 40, 60, 80 and 100 nodes as seeds. In terms of the *SIR* model, one can find that the disease cannot spread if the infected probability β is too small. The main reason is that the seeds have only a small probability to affect their neighbors. Conversely, when the infected probability is too high, all nodes will become infected state. This is meaningless for accuracy analysis. Therefore, we mainly consider the propagation ability of seeds at the threshold of infected probability [46], i.e., β=1/(<d>−1) and γ=1.

**Figure 11:**
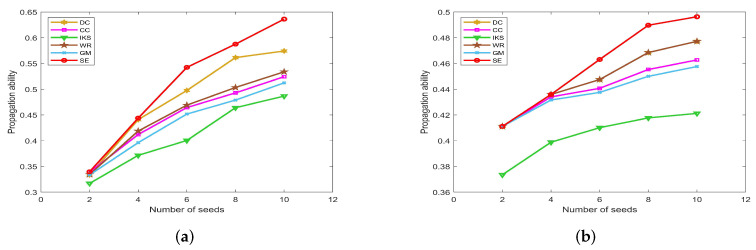
The propagation ability of seeds on (**a**) *CONT* and (**b**) *LESM* datasets.

**Figure 12:**
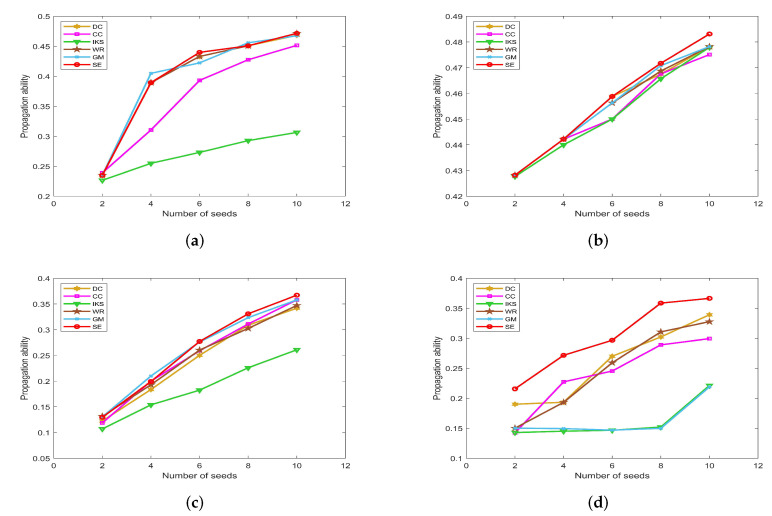
The propagation ability of seeds on (**a**) *POLB*; (**b**) *ADJN*; (**c**) *FOOT*; and (**d**) *NETS* datasets.

**Figure 13:**
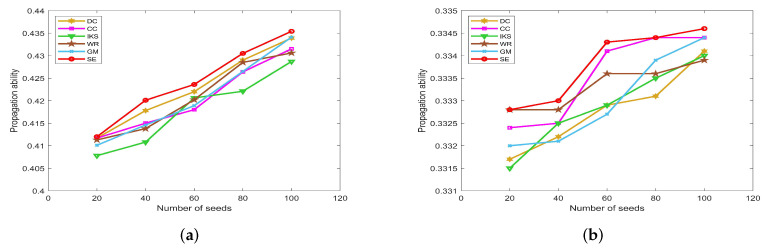
The propagation ability of seeds on (**a**) *EMAI* and (**b**) *HAMS* datasets.

Figure 11, Figure 12 and Figure 13 show the propagation ability of seeds obtained by six ranking methods on eight real-world datasets. From the results, one can find that the *SE* method can obtain a more accurate ranking sequence.

#### 5.4.1. On *CONT* and *LESM* Datasets

Figure 11 is the propagation ability of key nodes obtained by different methods on two small-scale datasets. Obviously, the *SE* method shows a more pronounced upward trend. That is to say, the top 10 key nodes obtained by the *SE* method have much higher propagation ability compared with other benchmark methods. Especially for the *CONT* dataset, the maximum propagation ability of *SE* method is 0.6361, which is 0.1495 higher than that of *IKS* method. Similarly, the maximum propagation ability of the *SE* method is 0.4963 on the *LESM* dataset, which is 0.0751 higher than that of the *IKS* method. Certainly, the *IKS* method performs poorly in most experiments. It can be seen from the previous experiments that the *IKS* method is not clear to identify the importance of different nodes. As a result, these nodes obtain the lowest propagation ability.

Figure 11a shows the fact that the *DC* method is obviously superior to the *WR* method on the *CONT* dataset. However, the propagation ability curves of *DC* and *WR* methods completely coincide on *LESM* dataset, as shown in Figure 11b. This is mainly because the top 10 nodes obtained by these two methods are the same. As can be seen from the foregoing discussion, both *DC* and *WR* methods consider the degree information of nodes. If the dataset contains many nodes with the same degree, the accuracy of *DC* and *WR* methods will decrease significantly. On the contrary, the *DC* and *WR* methods can obtain more accurate ranking sequences for datasets with significantly different degrees of nodes, such as the *LESM* dataset. However, in fact, the *SE* method takes the local and global structure information into account, and the accuracy of it is obviously better than that of *DC* and *WR* methods.

#### 5.4.2. On *POLB*, *ADJN*, *FOOT* and *NETS* Datasets

Figure 12 is the propagation ability of key nodes obtained by different methods on *POLB*, *ADJN*, *FOOT* and *NETS* datasets. On *POLB* dataset, the propagation ability curves of *DC*, *WR*, *GM* and *SE* methods all have an obvious upward trend, while the *IKS* is still the worst-performing method as shown in Figure 12a.

By observing Figure 12b,c, it can be found that the distribution of propagation ability curves is relatively dense. The main reason is that most of the methods obtain the same key nodes. For example, all methods treat nodes 18 and 3 as the top 2 key nodes except *IKS* method on *ADJN* dataset. Therefore, most methods achieve similar propagation ability curves. In this case, it should be pointed out that the *SE* method still has a slight advantage.

This advantage of the *SE* method is more significant on the *NETS* dataset. As can be seen from Figure 12d, it obtains the maximum propagation ability among all of the benchmark methods. Especially when the number of seeds is 8, the propagation ability of the *SE* method is 0.2087 higher than that of the *GM* method and 0.2065 higher than that of the *IKS* method. This means that the key nodes obtained by the *SE* method can infect 136 nodes, which is 79 higher than that of the *GM* method and 78 higher than that of the *IKS* method. Therefore, we can conclude that the ranking sequence obtained by the *SE* method is more accurate compared with other benchmark methods.

#### 5.4.3. On *EMAI* and *HAMS* Datasets

Figure 13 is the curves of propagation ability on *EMAI* and *HAMS* datasets. As the scale of the dataset increases, the number of seeds we selected also increases to 100. From Figure 13a, one can find that the *SE* method can maintain the obvious upward trend. One can find that the *SE* method outperforms the other benchmark methods after the number of seeds exceeds 20. Since the special structure of the *HAMS* dataset, the variation range of propagation ability is small. For this, the *SE* method still has a slight advantage compared with other methods as shown in Figure 13b. This further confirms that the *SE* method has higher ranking accuracy compared with other benchmark methods.

### 5.5. Computational Complexity Analysis

Given that G=(V,E) is a graph data with *n* nodes and *m* edges, and the proposed *SE* method includes two stages in the process of constructing the score function for nodes. Firstly, the computational complexity of calculating the local structure entropy is O(sn), where *s* is the number of connected components in *G*. Secondly, the computational complexity of calculating the global structure entropy is O(n). Therefore, the total computational complexity of *SE* method is O(sn+n)=O(n(s+1)).

Table 4 lists the computational complexity of the proposed *SE* method and other benchmark methods. One can find that the computational complexity of the *CC* method is O(nm), and that of the *GM* method is $O(n^{2})$ [47]. Due to *s* being the number of connected components after removing the target node, the value of *s* is far smaller than *m* and *n*. That is to say, the computational complexity of the *SE* method is much lower than that of the *CC* and *GM* methods. Although *DC* and *IKS* methods have the lowest computational complexity, their performance is far worse than that of other methods in previous experiments. In general, although the computational complexity of the *SE* method is in the middle position among all comparison methods, it can obtain better ranking results.

## 6. Conclusions

In order to further explore the influence of structure information on node importance, this paper has designed a structure entropy-based node importance ranking method. The score function of node importance is constructed from the perspective of node removal, which transformed the importance of nodes into the global structure entropy of graph data. After removing the target node, the local structural entropy of the connected component is calculated by using the degree information of nodes. Furthermore, the global structure entropy of graph data is constructed in terms of the number of connected components. A large number of experiments demonstrated that the proposed method is more advantageous in aspects of monotonicity, node distribution and ranking accuracy.

Although the proposed method has better performance on most datasets, it is not hard to see that this paper only discussed the undirected and unweighted graph data with less than 2000 nodes due to the limitation of the experimental platform. In our following studies, we will seek more resources to verify the performance of the proposed method on larger-scale graph data and other types of graph data.

## Figures and Tables

**Table 1:** The global structure entropy of top six nodes.

Order	1	2	3	4	5	6
Node	$v_{5}$	$v_{4}$	$v_{10}$	$v_{6}$	$v_{2}$	$v_{3}$
$SE(v_{i})$	20.7337	28.5322	29.7450	32.1098	36.9700	36.9700

**Table 2:** The topological statistical information of eight real-world networks.

Dataset	*n*	*m*	<d>	$d_{\max}$	cc
*CONT*	49	107	4.3673	8	0.4061
*LESM*	77	254	6.5974	36	0.4989
*POLB*	105	441	8.4000	25	0.4875
*ADJN*	112	425	7.5893	49	0.1898
*FOOT*	115	613	10.6609	12	0.4032
*NETS*	379	914	4.8232	34	0.7981
*EMAI*	1133	10,903	9.6230	71	0.2550
*HAMS*	1576	4032	5.1168	147	0.1312

**Table 3:** The M value of six ranking methods. The best results are highlighted in bold.

Dataset	M (*DC*)	M (*CC*)	M (*IKS*)	M (*WR*)	M (*GM*)	M (*SE*)
*CONT*	0.6973	0.9780	0.7942	0.9546	0.9966	**1.0000**
*LESM*	0.8147	0.9414	0.8134	0.9547	**0.9581**	**0.9581**
*POLB*	0.8252	0.9846	0.8382	0.9967	0.9996	**1.0000**
*ADJN*	0.8661	0.9837	0.8745	0.9961	0.9994	**0.9997**
*FOOT*	0.3636	0.9488	0.9419	0.9281	0.9985	**1.0000**
*NETS*	0.7642	0.9928	0.7607	0.9839	0.9949	**0.9953**
*EMAI*	0.8874	0.9988	0.8981	0.9977	**0.9999**	**0.9999**
*HAMS*	0.6263	0.6834	0.6292	0.6829	**0.6839**	**0.6839**

**Table 4:** The computational complexity of six ranking methods.

Method	*DC*	*CC*	*IKS*	*WR*	*GM*	*SE*
Complexity	O(n)	O(nm)	O(n)	O(m+n<d>)	$O(n^{2})$	O(n(s+1))

## Data Availability

Not applicable.
